# Epidemiology of isolated olecranon fractures: a detailed survey on a large sample of patients in a suburban area

**DOI:** 10.1016/j.jseint.2021.11.015

**Published:** 2022-01-06

**Authors:** Matteo Cantore, Vittorio Candela, Pasquale Sessa, Giuseppe Giannicola, Stefano Gumina

**Affiliations:** a Department of Anatomical, Histological, Forensic Medicine and Orthopaedics Sciences, Sapienza University of Rome, Istituto Clinico Ortopedico Traumatologico (ICOT), Latina, Italy; b Department of Orthopaedics and Traumatology, Azienda Ospedaliera San Camillo-Forlanini, Rome, Italy; c Department of Anatomical, Histological, Forensic Medicine and Orthopaedics Sciences, Sapienza University of Rome-Policlinico Umberto I, Rome, Italy

**Keywords:** Isolated olecranon fractures, Olecranon fractures epidemiology, Proximal forearm fractures, MAYO classification, AO classification, Proximal ulna trauma mechanisms

## Abstract

**Background:**

Literature lacks data concerning several epidemiologic aspects of isolated olecranon fractures (IOFs). The few studies that have analyzed this type of fracture show a low sample size and contradicting results.

**Methods:**

This retrospective study included 165 consecutive patients (82 men and 83 women) who sustained an IOF in the past 10 years. Participants who were aged <16 years or had a previous elbow fracture or had a fracture that involved other bones of the elbow joint were excluded. Data regarding age, sex, season, date, and fracture side were collected. As per the mechanism of injury, we arbitrarily distinguished 7 subgroups. IOFs were classified as per the Mayo and AO classifications using x-ray. Statistics were performed.

**Results:**

The patients’ mean age was 58.5 (standard deviation [SD], 21.3) years, and men and women were aged 48.1 (SD, 19.8) years and 67.9 (SD, 18.8) years, respectively. The most frequent fracture patterns were the MAYO 2A and the AO 2U1B1(d). Low-energy mechanisms caused simple dislocated-stable fractures, whereas high-energy mechanisms caused both simple and comminuted displaced-stable fractures. Significant differences in the trauma mechanism were found between male and female patients. The former fractures showed a bimodal distribution depending on the patients’ age group, whereas in women, the traumatic event was mainly represented by a low-energy mechanism. Overall, the most common cause of fracture was a low-energy accident. The seasonal distribution of fractures was different for male and female patients being more frequent in summer among young men and more frequent in winter among the elderly, both men and women. The left side was involved in 87 patients.

**Conclusion:**

IOFs occur equally in both genders, although with different age distribution. The most common fracture pattern was a simple displaced-stable fracture (MAYO 2A and AO 2U1B1[d]). Young men are more often subject to high-energy injuries that occur in road accidents, whereas with aging, they become more prone to fragility fractures as women. Female patients are usually older and are mostly affected by low-energy traumas as a fall from a standing height.

Olecranon fractures (OFs) represent approximately 10% of all elbow and 20% of proximal forearm fractures^5^^,^^10^ and are also the most common type of proximal ulna fracture.^5^^,^^11^ As per literature, the incidence of this type of fracture varies from 11.5 to 12 per 100,000 population,^5^^,^^6^ and it can be caused by various traumatic mechanisms, both direct and indirect, although the former seems more frequent.^1^ Many authors have studied the management and postoperative follow-up of these lesions^13^; however, very few studies have analyzed in depth the epidemiology of this fracture, and those studies that have analyzed show a low sample size and contradictory results.^5^^,^^6^^,^^11^ Data collected in two of the most representative studies about OF epidemiology differ greatly for sex, age, and mechanism of trauma. Duckworth et al^5^ studied 78 fractures of the proximal ulna, of which 64 fractures are of the olecranon and found that these were more frequent among elderly women who reported a low-energy fall at home or in the street. On the contrary, Nieto et al,^11^ who studied 98 isolated OFs (IOFs), found that patients were mainly young and active men, and the most common type of injury was road traffic accidents.

The goal of this study is to analyze a large number of patients with IOF that occurred in the last 10 years in a suburban area and to provide a detailed epidemiologic survey.

## Materials and methods

We have conducted a retrospective search of the IOFs that were registered in the database of the local emergency department which serves an area of more than 550,000 inhabitants. To carry out the research, we have used the International Statistical Classification of Diseases and Related Health Problems, Ninth Version codes. In particular, we have used the codes for “closed olecranon fractures” (81301) and “open olecranon fractures” (81311). The database was maintained on a digital platform.

Two authors (M.C. and C.V.) collected information regarding sex, age, date of fracture, mechanism of injury, and fracture side. Trauma mechanisms were divided in 7 subgroups: (1) low-energy trauma occurred at home, (2) low-energy trauma occurred in an urban environment (when walking and running), (3) work-related injuries, (4) trauma resulting from direct hit, (5) high-energy trauma resulting from high fall, (6) sports trauma, and (7) high-energy trauma resulting from car, motorcycle, public transport, and pedestrian accidents. To facilitate the statistical analysis, mechanisms 1 and 2 were grouped as low-energy mechanisms of trauma, whereas mechanisms 5, 6, and 7 were grouped as high-energy mechanisms of trauma.

To evaluate the correlation among parameters, patients were divided into 3 subgroups as per age: (1) patients aged between 16 and 45 years, (2) patients aged between 46 and 75 years, and (3) patients older than 76 years.

All fractures were assessed using x-ray standard elbow trauma series consisting of a true anteroposterior view, a lateral view, and an oblique view. In three cases (1.8%) which showed a stable fracture, the diagnosis was made with computed tomography (CT) scans because simple x-rays were dubious.

A total of 110 patients with an OF were excluded from the study. These included patients younger than 16 years and those who presented a fractured capitellum or coronoid process or previous elbow surgical treatments.

IOFs were classified using the MAYO and the AO classification systems. Each fracture was classified twice by 3 authors (G.S., C.M., and C.V.) at a 3-month interval. Intrarater reliability and inter-rater reliability were statistically assessed.

As per our country laws, this study does not need the ethical committee approval.

### Statistical analysis

A descriptive analysis was performed for all examined parameters. Exact Fisher F-test was used to identify any difference between age, mechanism of trauma, time of the year of the injury, and fracture pattern when analyzing male and female patients, both together and separately.

Intrarater reliability and inter-rater reliability were studied with κ statistics according to Cohen. The κ values for intrarater reliability were calculated for each observer before the mean κ value was obtained. The κ values for inter-rater reliability were calculated for each possible pair of the 3 observers before the mean κ value was obtained. The Landis and Koch criteria were used to assess the obtained data. The κ values are reported as mean and 95% confidence interval (CI).

The level of significance was set at alpha ≤0.05. IBM SPSS Statistics for Windows 20.0 software (IBM, Armonk, NY, USA) was used.

## Results

A total of 165 consecutive patients managed in the emergency department of our hospital for IOF from January 30, 2011, to January 30, 2021, were selected of a total of 32,400 fractures. The number of male patients was 82 (49.7%), whereas female patients were 83 (50.3%).

The patients’ mean age was 58.5 (standard deviation [SD], 21.3) years, and men and women were aged 48.1 (SD, 19.8) years and 67.9 (SD, 18.8) years, respectively. The left side was more frequently involved (87 cases, 52.7%) (*P* = .53).

All 165 IOFs were classified using the MAYO^2^^,^^14^ and the AO/OTA (Arbeitsgemenschaft Für Osteosynthesefragen/Orthopaedic Trauma Association)^8^^,^^9^ classification systems. The former classification divides fractures into three types (I, II, and III) depending on the stability and the displacement shown on the X-rays, and each type is further divided into uncomminuted (A) and comminuted (B). The AO classification assigns a number to every bone of the body (ulna is 2U) and an additional number to identify if the fracture involves the proximal (1), diaphyseal (2), or distal (3) segment of the bone. The end segment fractures are divided into extra-articular (A), partial articular (B), and complete articular (C). An ulterior qualification can be added depending on the characteristics of the fracture varying in the different bones.

**Figure 1:**
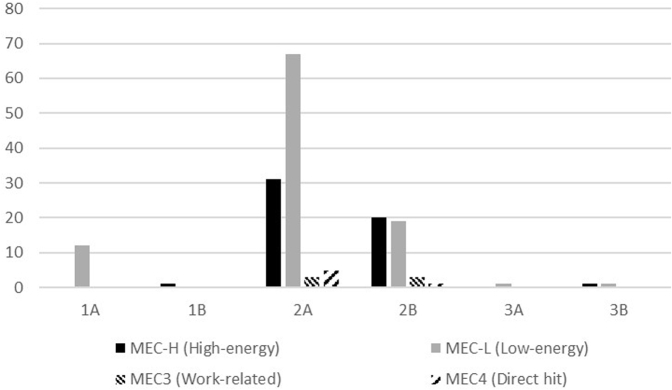
Fracture patterns as per the MAYO classification.

The most frequent patterns of fracture as per the MAYO system were 2A (64.24%), 2B (26.06%), 1A (7.27%), 3B (1.21%), 3A (0.61%), and 1B (0.61%), whereas for the AO system, the most frequent patterns were 2U1B1(d) (73.94%) and 2U1B1(e) (26.06%). Only 3 patients had an open fracture, accounting for 1.8% of IOFs. When considering all patients, MAYO IIa fractures were more frequently caused by low-energy mechanisms, whereas type 2B fractures were a consequence of both low-energy and high-energy injuries (*P* = .047) (Fig. 1). Among male patients, type 2B fractures were observed especially in patients aged between 16 and 45 years (*P* = .021), and high-energy mechanisms of trauma were associated with MAYO 2B (*P* = .01) and AO 2U1B1(e) (*P* = .031) fractures. Elbow dislocation was observed in 2 patients (1.21%) and was associated with a 2A (2U1B1d) and 3A (2U1B1d) fracture pattern.

**Figure 2:**
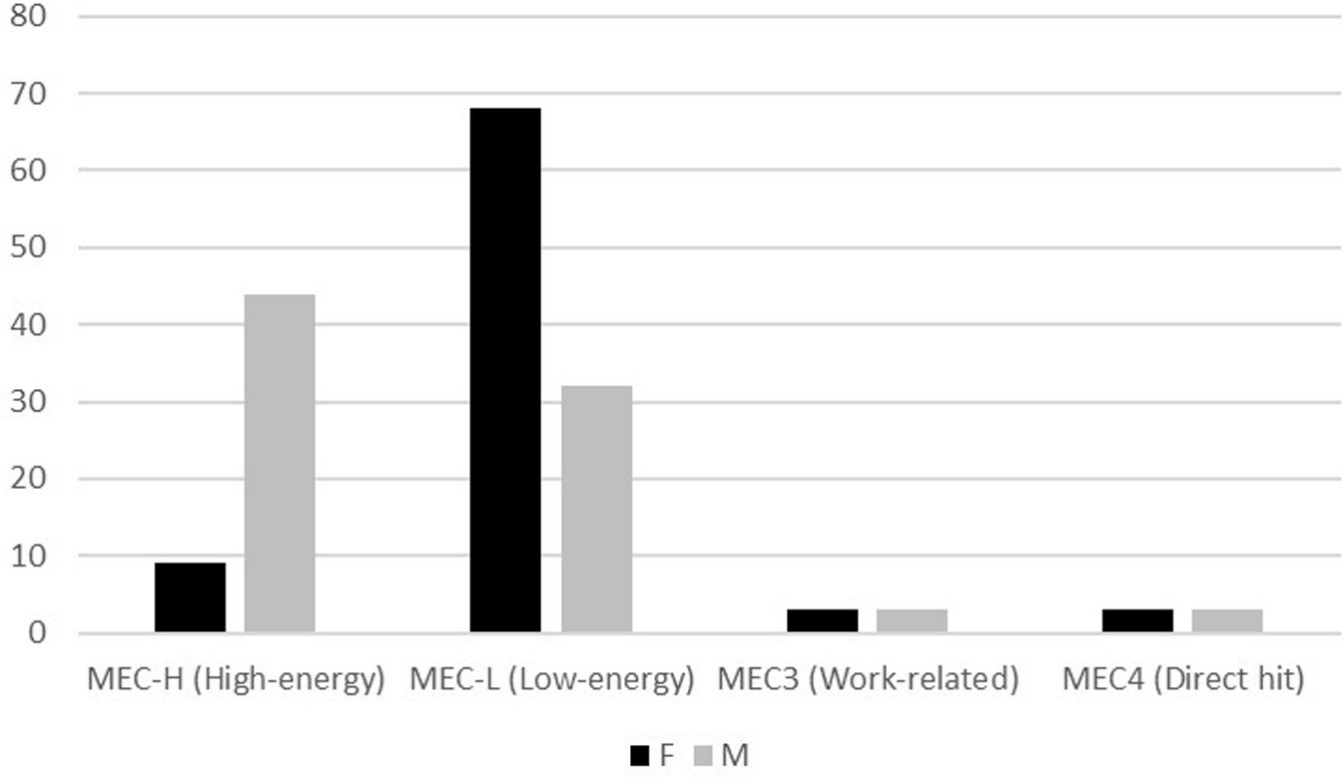
Distribution of isolated olecranon fractures in both genders as per the mechanism of trauma.

Figure 2 shows the distribution of IOFs in men and women as per the trauma mechanism. In women, the most frequent type of injury was represented by low-energy traumatic events, whereas in men, by high-energy mechanisms (*P* < .001). When considering age groups, the most frequent cause of injury was a high-energy trauma in patients aged 17-75 years and a low-energy trauma in older patients (*P* < .001).

**Figure 3:**
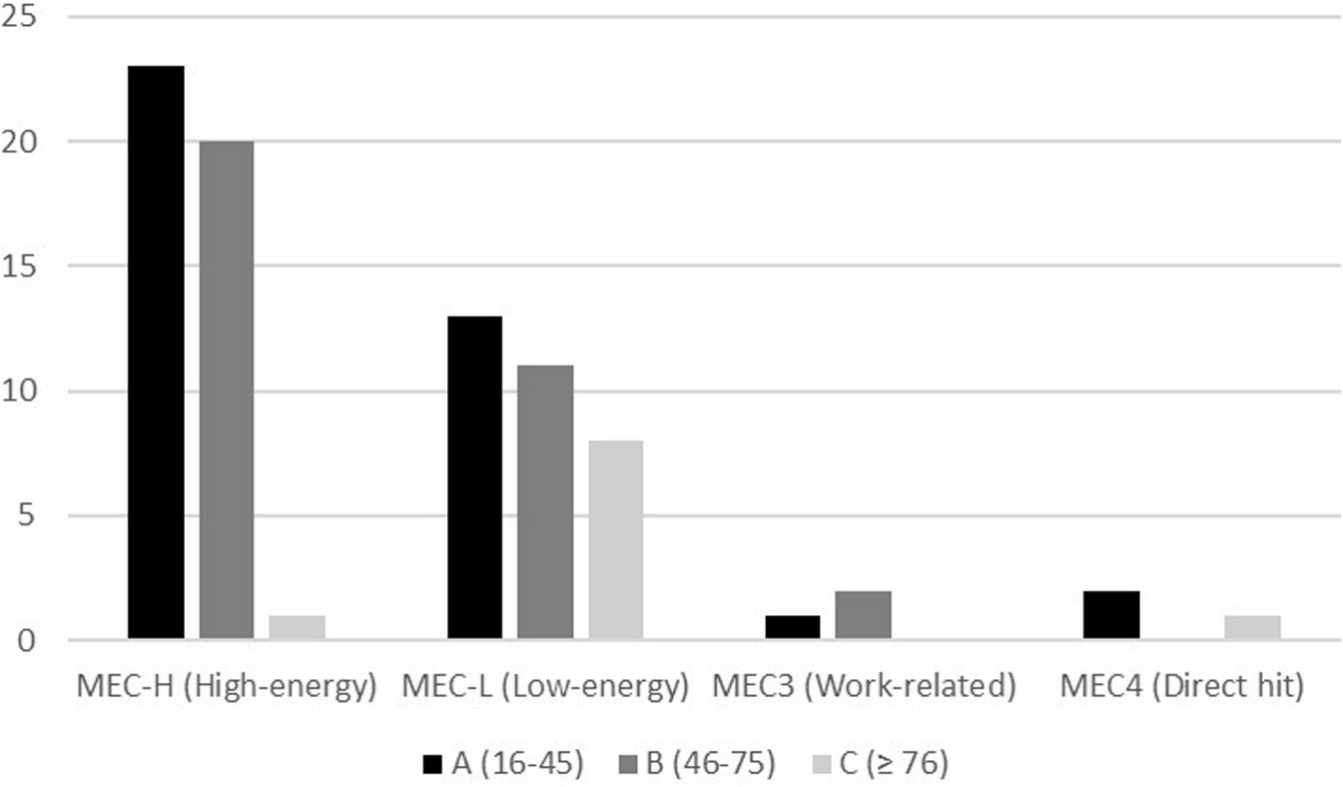
Distribution of fractures in men as per the mechanism of trauma.

Male patients showed a bimodal distribution of the fracture mechanism. A high-energy mechanism (5, 6, and 7) was observed in 59.7% of patients between 17 and 75 years old, whereas a low-energy mechanism (1 and 2) was found in 80% of patients older than 76 years (*P* = .02) (Fig. 3).

The trauma mechanism in female patients was mainly represented by low-energy injuries (1 and 2), involving 68.1% of patients aged <75 years and 97.4% of patients aged >75 years (*P* < .001) (Table I).

**Table I:** Distribution of olecranon fractures in women as per age group and mechanism of trauma.

		Mechanism of trauma				Total
		MEC-H	MEC-L	MEC3	MEC4	
Age group	A	4	7	2	2	15
	B	5	23	1	0	29
	C	0	38	0	1	39
Total		9	68	3	3	83

*MEC-H*, high-energy mechanism of trauma; *MEC-L*, low-energy mechanism of trauma.

**Figure 4:**
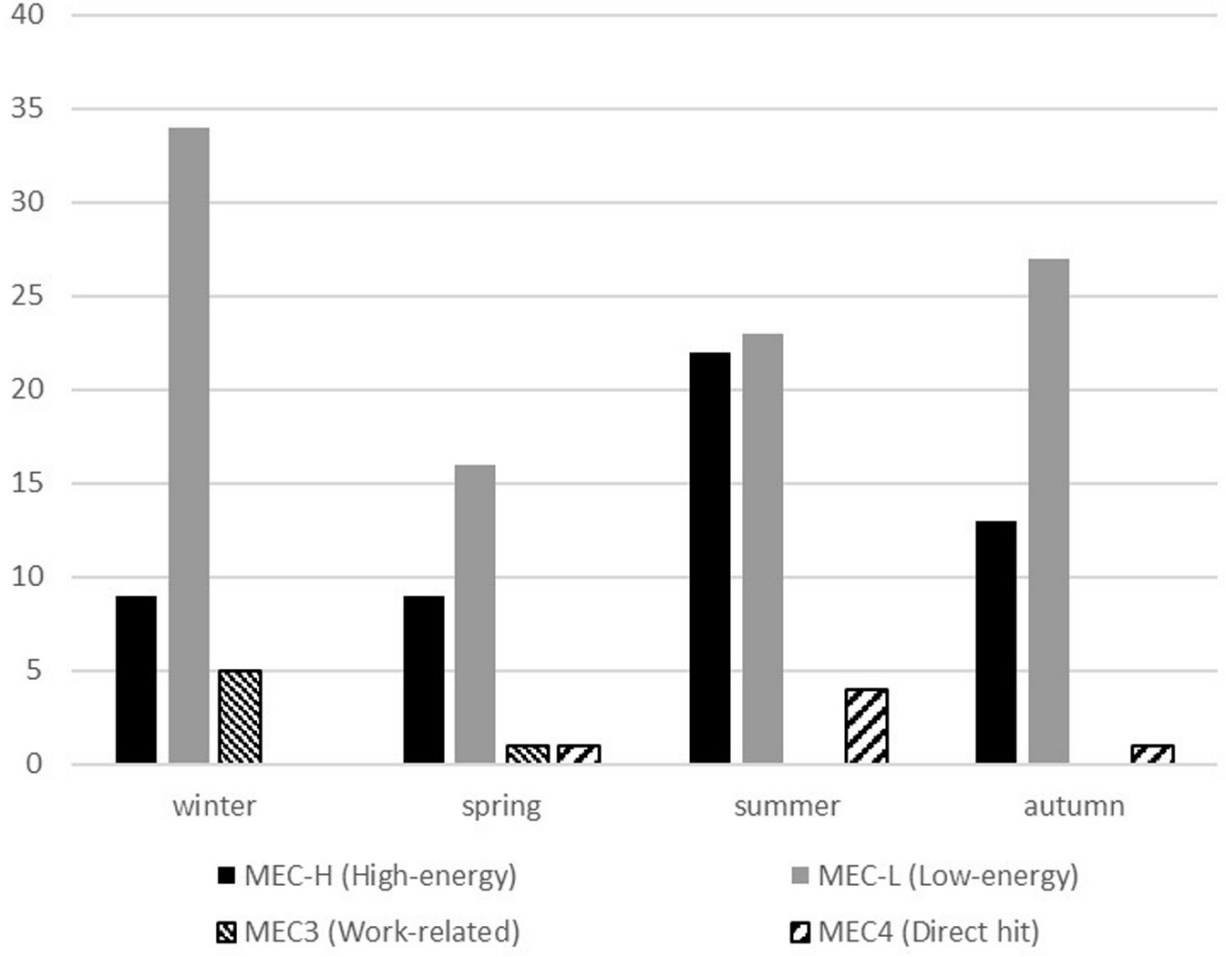
Seasonal distribution of injuries as per the mechanism of trauma.

**Figure 5:**
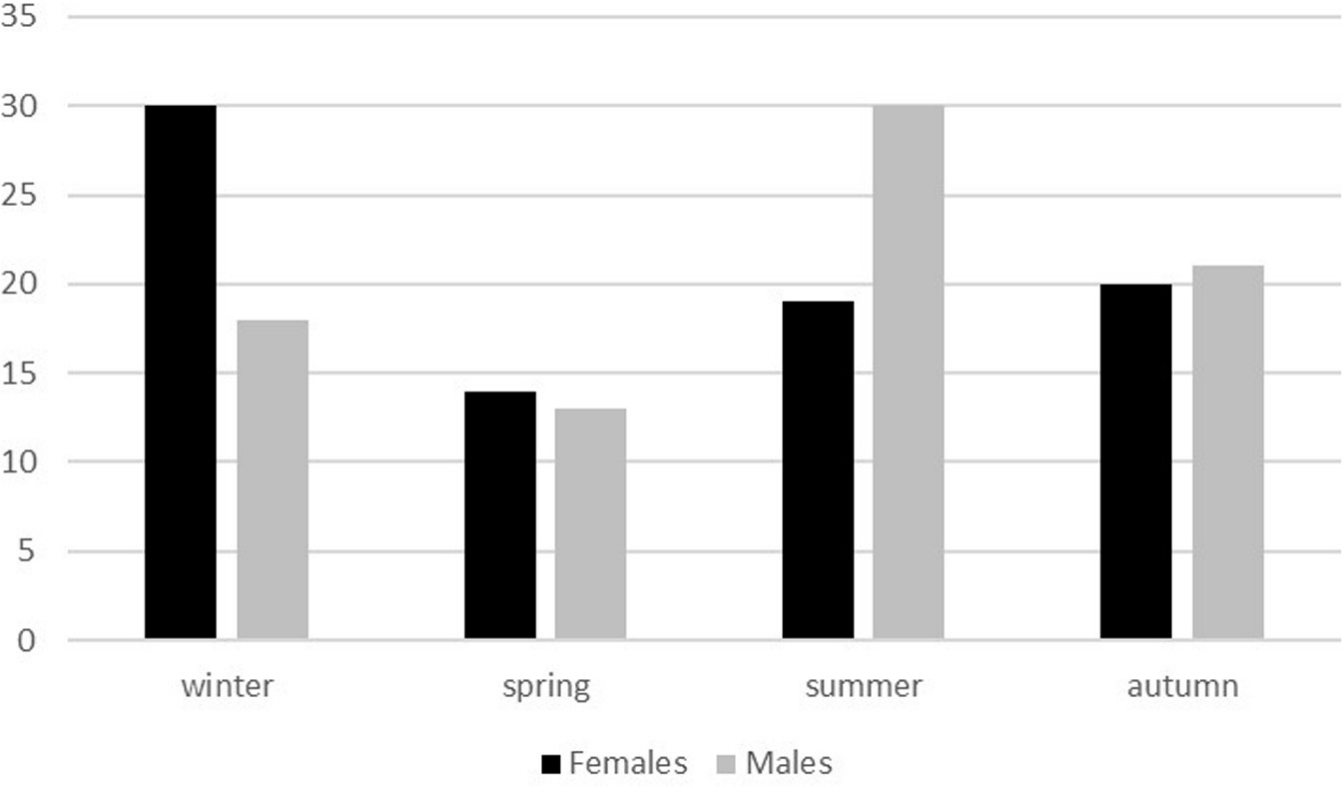
Seasonal distribution of olecranon fractures in men and women.

Analyzing the seasonal distribution of fractures, low-energy mechanisms appeared well-represented all year round, but especially in winter, whereas high-energy injuries mainly occurred in summer (*P* = .007) (Fig. 4). The seasonal distribution of IOFs in men and women is shown in Figure 5. IOFs occurred more frequently in winter and summer seasons, although with an inverse distribution between men and women. In men, high-energy and low-energy fractures were more frequent in summer and winter, respectively (*P* = .03). During the other periods of the year, the prevalence of IOFs was lower (<17%). No significant differences were found between male and female patients when considering bimesters or seasons (*P* = .33 and *P* = .14).

No significant differences were observed when analyzing the day of the week.

The mean κ value for intraobserver reliability assessment was 0.97 (95% CI, 0.96-0.98), whereas the mean value for interobserver reliability was 0.93 (95% CI, 0.91-0.95).

## Discussion

To date, few studies have analyzed the epidemiology of IOFs. In particular, how the traumatic mechanism is correlated with age, sex, seasons, and weekdays has not been studied in detail. In our study, the goal was to further investigate all these aspects to achieve a better knowledge of this fracture. IOFs represent the most common fractures of the elbow.^5^^,^^11^ In 2002, Karlsson et al^6^ in a follow-up study on just 73 patients found that the incidence of this type of fracture is of 11.5 per 100,000 population, which was comparable with the findings of Duckworth et al^5^ that in a retrospective study on 64 patients found an incidence of 12 per 100,000 population.

As per literature, IOFs are mostly fragility fractures caused by low-energy falls and affect women and elderly patients more frequently;^3^, ^4^, ^5^, ^6^^,^^16^ however, in the study by Niéto et al,^11^ the main mechanism of trauma was represented by high-energy road traffic accidents and overall men were more affected than women. As in Duckworth’s study,^5^ we found that in women, the mean age (67.9 years) was considerably higher than in men (mean age: 48.1 years), and this may be due to the longer life expectancy and to the higher incidence of osteoporosis.

Most of the authors who studied OFs used the MAYO classification system.^2^^,^^14^ In 2014, Tamaoki et al^15^ compared different classification systems for OFs and concluded that the best method for both interobserver and intraobserver reliability was the MAYO and the AO classification systems. In our study, we have decided to use both these methods using x-rays and CT scans where possible.

Overall, the most frequent patterns that we found were the MAYO 2A and the AO 2U1B1(d) (64.2% and 74%, respectively) as observed in other studies. The trauma mechanism influenced the fracture pattern, especially when as per the MAYO classification. In fact, we found a statistically significant difference among the different patterns, and more precisely low-energy mechanisms caused simple dislocated-stable fractures (2A), whereas high-energy mechanisms caused comminuted displaced-stable fractures (2B and 2U1B1[d]).

Regarding the mechanism of trauma, IOFs are caused by both direct and indirect trauma, although the former is more common. In 1995, Amis et al^1^ conducted a study on 40 cadaveric elbows that were mounted onto a purpose-built impact loading rig and were then fractured with a swinging impactor pendulum; the authors found that the olecranon was easily fractured when directly impacted in the 60° to 110° arc of flexion. We found a statistical difference between men and women. High-energy mechanisms such as sports accidents, high falls, and motor vehicle collisions (MVCs) were overall the most frequent cause of IOFs in men, whereas a low-energy mechanism as a fall from a standing height at home or in the street represented the most common mechanism among women. No differences in prevalence were found between men and women when considering workplace and direct hit injuries. These results may be related to several aspects such as the longer time spent at home, longer life expectancy, reduced muscular strength, gait disorders, and higher incidence of severe osteoporosis^7^ that characterize elderly women. In men, as observed in other studies regarding upper extremity fractures,^12^ a bimodal age distribution as per the trauma mechanism was detected, with almost 60% of patients aged 17-75 years who were injured by a high-energy mechanism (MEC-5, 6, and 7), whereas a low-energy mechanism (MEC-1 and 2) was found in 80% (n = 8) of patients older than 76 years. These results may be related to the fact that younger patients are more frequently involved in MVCs, especially motorcycle accidents (34% of all high-energy injuries and 55.5% of all MVCs), whereas older patients are more prone to low-energy traumas probably as a consequence of the lower bone density associated with aging. Because in both male and female elderly subjects, these fractures are mainly caused by low-energy mechanisms, it is reasonable to consider them as primary fractures related to an underlying osteoporotic condition which may require adequate preventative therapy to reduce the risk of future and more serious fragility fractures.

When considering the mechanism and the time of the year, high-energy trauma showed a greater frequency in summer as the result of a greater use of motorcycles and bicycles in this season, whereas winter was mainly characterized by low-energy injuries. This is particularly true for male patients, and this may be related to the different age distribution of injured patients during the year.

In our study, we found that IOFs were more frequent in winter and summer seasons, although women were more affected in the former and men were more affected in the latter. These data may be related to the weather conditions and to people’s habits. Probably in summer, people travel more, resulting in an increased number of accidents, whereas in winter, the fewer hours of sunlight could increase the risk of falls in both domestic and urban environments.

Our study presents one main limit represented by the use of conventional x-rays (98.2% of cases). CT scan was considered a second-stage diagnostic tool prescribed only in case of diagnostic doubts.

## Conclusion

This epidemiologic study on a large sample demonstrated that IOFs are equally frequent in men and women and that the main patterns of fracture in both genders and in all age groups are MAYO 2A and AO 2U1B1(d). Female patients showed a higher mean age and an increasing prevalence of fractures with aging. Male patients presented a lower mean age and a bimodal distribution depending on the mechanism of trauma.

This study also showed that the mechanism of fracture and the season in which it occurred are different for male and female patients. In the former, the main mechanism is represented by a high-energy injury that occurred mainly in summer, whereas in the latter, the main cause of injury was a low-energy trauma that happened in winter.

## Disclaimers

Funding: No funding was disclosed by the authors.

Conflicts of interest: The authors, their immediate families, and any research foundation with which they are affiliated have not received any financial payments or other benefits from any commercial entity related to the subject of this article.
